# Results of 3,668 primary total hip replacements for primary osteoarthritis in patients under the age of 55 years: A follow-up of a previous report from the Finnish Arthroplasty Register

**DOI:** 10.3109/17453674.2011.618908

**Published:** 2011-11-24

**Authors:** Keijo T Mäkelä, Antti Eskelinen, Pekka Pulkkinen, Pekka Paavolainen, Ville Remes

**Affiliations:** ^1^Department of Orthopaedics and Traumatology, Turku University Central Hospital; ^2^COXA Hospital for Joint Replacement, Tampere; ^3^Department of Public Health, University of Helsinki; ^4^ORTON Orthopaedic Hospital, Invalid Foundation, Helsinki; ^5^Department of Orthopaedics and Traumatology, Peijas, Helsinki University Central Hospital, Finland

## Abstract

**Background and purpose:**

In a previous study based on the Finnish Arthroplasty Register, the survival of cementless stems was better than that of cemented stems in younger patients. However, the survival of cementless cups was poor due to osteolysis. In the present study, we analyzed population-based survival rates of the cemented and cementless total hip replacements in patients under the age of 55 years with primary osteoarthritis in Finland.

**Patients and methods:**

3,668 implants fulfilled our inclusion criteria. The previous data included years 1980–2001, whereas the current study includes years 1987–2006. The implants were classified in 3 groups: (1) implants with a cementless, straight, proximally circumferentially porous-coated stem and a porous-coated press-fit cup (cementless group 1); (2) implants with a cementless, anatomic, proximally circumferentially porous-coated stem, with or without hydroxyapatite, and a porous-coated press-fit cup with or without hydroxyapatite (cementless group 2); and (3) a cemented stem combined with a cemented all-polyethylene cup (the cemented group). Analyses were performed separately for 2 time periods: those operated 1987–1996 and those operated 1997–2006.

**Results:**

The 15-year survival for any reason of cementless total hip replacement (THR) group 1 operated on 1987–1996 (62%; 95% CI: 57–67) and cementless group 2 (58%; CI: 52–66) operated on during the same time period was worse than that of cemented THRs (71%; CI: 62–80), although the difference was not statistically significant. The revision risk for aseptic loosening of cementless stem group 1 operated on 1987–1996 (0.49; CI: 0.32–0.74) was lower than that for aseptic loosening of cemented stems (p = 0.001).

**Interpretation:**

Excessive wear of the polyethylene liner resulted in numerous revisions of modular cementless cups. The outcomes of total hip arthroplasty appear to have been relatively unsatisfactory for younger patients in Finland.

Only a few register-based studies have yielded results of THA for primary osteoarthritis in younger patients at a population-based level (Havelin et al. 2000, Malchau et al. 2002, Eskelinen et al. 2005, 2006). In patients under the age of 55 years, data from population-based studies have suggested that the survival of cementless, proximally porous-coated stems can be as good as that of cemented stems (Havelin et al. 2000, Eskelinen et al. 2005). However, it has not been clear whether cementless cups perform as well as cemented cups in younger patients.

On the basis of the Finnish Arthroplasty Register, we evaluated population-based data on the survival of primary total hip replacements performed for primary osteoarthritis in patients under the age of 55 years. The data including years 1980–2001 has been published earlier (Eskelinen et al. 2005), whereas the current study includes data from 1987 to 2006.

## Patients and methods

Since 1980, data on total hip replacements have been collected by the Finnish Arthroplasty Register (Paavolainen et al. 1991, Puolakka et al. 2001). Healthcare authorities, institutions, and orthopedic units in Finland are obliged to provide the National Agency for Medicines with information that is essential for monitoring past and current trends for the efficacious use of materials, approaches, and designs used in orthopedic medicine. The coverage in the Finnish Arthroplasty Register was initially analyzed for the period from 1994 to 1995 by comparing its data with those of the discharge registers of participating hospitals. 90% of all implantations actually performed were reported to the register (Puolakka et al. 2001). Since 1995, the data in the register have been compared with those in hospital discharge registers every few years. Currently, 98% of implantations are recorded in the Finnish Arthroplasty Register (Peltola 2009).

### Inclusion and exclusion criteria

Only patients under the age of 55 years at the time of the primary operation were included. In order to eliminate the effect of diagnosis as a confounding factor, only patients with primary osteoarthritis as a recorded indication for operation were included. Only total hip replacements (cup and stem combinations) that had been used in more than 10 operations during the study period were included in the present study (Havelin et al. 1995a, b). Prosthetic components with well-documented poor results, e.g. cementless, smooth-threaded cups (Engh et al. 1990, Tallroth et al. 1993, Simank et al. 1997, Eskelinen et al. 2005), and implants that did not fit into any of the groups of interest (see below) were excluded. The data of cement brands used have been included in the Finnish Register from 1996 onward. We excluded cement brands with known poor results, such as Boneloc and CMW, from the study.

### Implant group analysis

The success rate of different implant groups was analyzed. All hip replacements included were classified into the following groups: (1) a cementless, straight, proximally circumferentially porous-coated stem with a porous-coated pressfit cup (cementless group 1); (2) a cementless, anatomic, proximally circumferentially porous-coated stem with or without hydroxyapatite, and a porous-coated press-fit cup with or without hydroxyapatite (cementless group 2); and (3) a cemented total hip replacement (a cemented stem combined with a cemented all-polyethylene cup).

Femoral components of the total hip replacements included were separately classified into 3 stem groups: (1) cementless, straight, proximally circumferentially porous-coated; (2) cementless, anatomic, proximally circumferentially porous-coated with or without hydroxyapatite (fit and fill); and (3) cemented.

Acetabular components of the total hip replacements included were also separately classified into 3 cup groups: (1) cementless, press-fit porous-coated; (2) cementless, hydroxyapatite-coated; and (3) cemented all-polyethylene.

### Study population

During the whole study period (1987–2006), 97,164 primary THRs were performed in Finland. Of these operations, 13,115 (13%) were performed on patients under the age of 55 years. Primary osteoarthritis was an indication in 50% (n = 6,578) of these operations. After exclusion of implants according to our study criteria, 3,668 total hip replacements were included in the final analysis (Table 1).

**Table 1.** Demographic data on total hip replacements analyzed in the study

Total hip replacement group	Number of operations	Follow-up (year) **^a^**	Age (year) **^a^**	Females (%)	Number of hospitals
1987–1996					
Cementless group #1	767	10.9 (0–18)	48 (24–54)	45	41
Cementless group #2	419	11.3 (0–20)	49 (27–54)	47	40
Cemented THR	140	11.6 (0–20)	50 (19–54)	56	31
Subtotal	1,326				
1997–2006					
Cementless group #1	1,632	4.5 (0–10)	50 (16–54)	47	59
Cementless group #2	534	5.2 (0–10)	50 (25–54)	48	38
Cemented THR	176	4.9 (0–10)	51 (26–54)	54	30
Subtotal	2,342				
Total	3,668				

**^a^** mean (range)

### Primary operations

During the study period, 96 different stem designs were used in patients who were younger than 55 years old and who had primary osteoarthritis. Of these stem designs, 47 were used in fewer than 10 operations. Cementless stems were used in 73% of the primary operations during the follow-up period. In patients who were less than 55 years old and had primary osteoarthritis, 93 different cup designs were used during the study period. Of these, 35 were used in fewer than 10 operations. Cementless cups were used in 90% of the primary operations during the follow-up period (Table 1).

### Revision operations

Revisions were linked to the primary operation using the unique personal identity number assigned to each resident of Finland. Revision is defined as either exchange or removal of the cup and/or stem, or exchange of the liner. Infections are mainly treated with 2-stage revision in Finland. Only the first operation, i.e. removal of the prosthesis, is recorded as first revision in the Finnish Arthroplasty Register. The second operation, i.e. re-replacement, is recorded as a re-revision and was not included in the study. During the study period, 502 revision operations were performed for patients in the study group (Table 2).

**Table 2.** Reasons for revision

A	B	C	D	E	F	G	H	I	J	K	L
1987–1996											
Cementless group #1	767	35	36	3	6	14	10	12	5	92	213
Cementless group #2	419	19	56	13	1	5	3	7	1	42	137
Cemented THR	140	14	12	7	0	1	0	1	0	0	35
1997–2006											
Cementless group #1	1,632	8	7	9	9	21	11	0	8	15	88
Cementless group #2	534	1	4	4	1	3	0	2	1	5	21
Cemented THR	176	3	1	0	2	2	0	0	0	0	8
Total	3,668	80	116	36	19	46	24	22	15	154	502

A Total hip replacement group cementless group #1: a cementless, straight proximally porous-coated stem and a modular cementless press-fit porous-coated cup; cementless group #2: a cementless, anatomic proximally porous and/or hydroxyapatite-coated stem and a modular press-fit and/or hydroxyapatite-coated cup. B Number of operations C Aseptic loosening (both) D Aseptic loosening (cup) E Aseptic loosening (stem) F Infection G Dislocation H Malposition I Fracture of the prosthesis J Periprosthetic fracture H Other reasons (including, for example, liner revisions due to excessive wear.) LTotal

### Statistics

The endpoint for survival was defined as revision when either one component (including liner and femoral head) or the whole implant was removed or exchanged. Both revision for any reason and revision for aseptic loosening served separately as endpoints. Revisions for any reason included (in addition to revisions for aseptic loosening of the cup and/or the stem) revisions for infection, dislocation, malposition, periprosthetic fracture, and fracture of the prosthesis, and other causes (including exchange of liner). The data from the study period 1987–2006 were analyzed in 2 separate time periods, 1987–1996 and 1997–2006, to have comparable follow-up periods in both study groups. Kaplan-Meier survival data were used to predict survival of implants at 5, 10, and 15 years. At each follow-up time point, survival rates were published only for implants with more than 20 patients at risk (Dorey 2004). Survival data obtained by Kaplan-Meier analysis were compared using the log-rank test. Patients who died or emigrated from Finland during the follow-up period were excluded at that point. The Cox multiple regression model was used to study differences between groups and to adjust for potential confounding factors. The factors studied with the Cox model were implant groups, age, and sex. Departures from the proportional hazards assumption were evaluated by visual inspection of the Cox curves. The follow-up period was divided into 2 parts to avoid crossing curves. In the current study, bilateral observations were included in the analysis. The bias introduced from neglecting bilateral prostheses is minute (Robertsson and Ranstam 2003).

When stem groups were analyzed with the Cox model, cemented stems with well-documented good long-term results (Havelin et al. 2000, Malchau et al. 2002, Williams et al. 2002, Callaghan et al. 2004, Buckwalter et al. 2006) served as the reference group. Similarly, all-polyethylene cemented cups (Havelin et al. 2000, Raber et al. 2001, Malchau et al. 2002, Sanchez-Sotelo et al. 2002, Williams et al. 2002, Callaghan et al. 2004, Della Valle et al. 2004, Buckwalter et al. 2006) served as the reference group on the acetabular side, and cemented total hip replacements (Havelin et al. 2000, Malchau et al. 2002, Williams et al. 2002, Issack et al. 2003, Callaghan et al. 2004, Della Valle et al. 2004, Buckwalter et al. 2006) served as the reference group in analyses of total hip replacements. When the effects of age and sex on implant survival were analyzed with the Cox model, adjustment was also made for implant groups (Furnes et al. 2001). Cox regression analyses provided estimates of survival probabilities and adjusted risk ratios for revision. Estimates from Cox analyses were used to construct adjusted survival curves at mean values of the risk factors. The Wald test was used to calculate p-values for data obtained from the Cox multiple regression analysis. Differences between groups were considered to be statistically significant if the p-values were less than 0.05 in a two-tailed test.

## Results

### Survival of THRs for any reason

**Figure 1.**
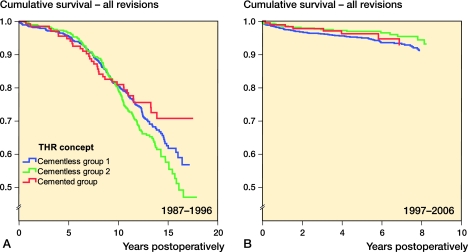
Cox-adjusted survival curves of 3,668 total hip replacements in patients aged less than 55 years with implant group as the strata factor. The endpoint was defined as revision of the stem and/or the cup for any reason. Adjustment was made for age and gender. A. THRs performed 1987–1996. Cementless group #2 had a significantly worse overall survival than the reference group of cemented hip replacements. B. THRs performed 1997–2006. The differences in survival rates between the THR groups were not statistically significant.

The 15-year survival for any reason for the cementless group 1 operated on 1987–1996 (62%, CI: 57–67) and cementless group 2 (58%, CI: 52–66) operated on during the same time period was worse than that of cemented THRs (71%, CI: 62–80), although the difference was not statistically significant (Table 3). The 10-year survival of cementless group 1 operated 1997–2006 was no better than that of the same group operated 1987–1996. In Cox regression analysis, there were no statistically significant differences in revision risk between the groups otherwise, but the revision risk for cementless group 2 operated 1987–1996 was higher than that for the cemented THRs operated during the same period (RR = 1.6; CI: 1.1-2.3) (p = 0.02) (Table 3, Figure 1).

**Table 3.** Survival of THR groups. The endpoint was defined as revision for any reason. 5-, 10-, and 15-year survival rates were obtained from the Kaplan-Meier analysis

A	B	C	D	E	F	G	H	I	J
1987–1996									
Cementless modular #1	767	722	95 (94–97)	595	80 (77–83)	108	62 (57–67)	1.30 (0.90–1.88)	0.2
Cementless modular #2	419	397	97 (95–98)	329	80 (76–83)	78	58 (52–64)	1.56 (1.07–2.27)	0.02
Cemented THR	140	128	95 (91–99)	105	81 (74–88)	33	71 (62–80)	1.0	–
1997–2006									
Cementless modular #1	1,632	775	95 (94–96)	61	79 (62–96)	0	–	1.27 (0.62–2.63)	0.5
Cementless modular #2	534	334	97 (95–99)	19	–	0	–	0.83 (0.37–1.87)	0.6
Cemented THR	176	93	96 (93–99)	3	–	0	–	1.0	
Total	3,668								

A Total hip replacement group cementless modular #1: a cementless, straight proximally porous-coated stem and a cementless press-fit porous-coated cup. cementless modular #2: a cementless, anatomic proximally porous- and/or hydroxyapatite-coated stem and a press-fit and/or hydroxyapatite-coated cup. B Number of primary operations C At risk at 5 years D 5-year survival (95% CI) E At risk at 10 years F 10-year survival (95% CI) G At risk at 15 years H 15-year survival (95% CI) I Risk ratio of revision (95% CI) from the Cox regression analysis (other groups were compared with the cemented total hip replacements; adjustment made for age and sex) J p-value

### Survival of THRs for aseptic loosening

**Figure 2.**
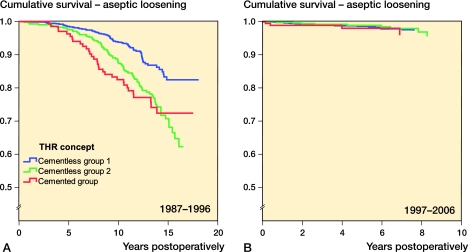
Cox-adjusted survival curves of 3,668 total hip replacements in patients aged less than 55 years with implant group as the strata factor. The endpoint was defined as revision of the stem and/or the cup for aseptic loosening. Adjustment was made for age and gender. A. THRs performed 1987–1996. Cementless group #1 had a significantly better overall survival than the reference group of cemented THRs. B. THRs performed 1997–2006. The differences in survival rates between the THR groups were not statistically significant.

In the Cox regression analysis, cementless group 1 had a lower risk of revision than the cemented THRs (RR = 0.5; CI: 0.32–0.74) (p = 0.001) (Table 4 and Figure 2). The 10-year survival of cementless group 1 operated 1997–2006 was better than that of the same group operated 1987–1996 (Table 4).

**Table 4.** Survival of THR groups. The end point was defined as revision due to aseptic loosening of the cup and/or the stem. 5-, 10-, and 15-year survival rates were obtained from the Kaplan-Meier analysis

A	B	C	D	E	F	G	H	I	J
1987–1996									
Cementless modular #1	767	719	99 (98–99)	589	94 (92–96)	107	82 (77–87)	0.49 (0.32–0.74)	0.001
Cementless modular #2	419	397	98 (96–99)	325	88 (84–91)	78	70 (64–77)	1.00 (0.66–1.50)	1.0
Cemented THR	140	128	96 (93–100)	105	82 (76–89)	33	72 (63–81)	1.0	–
1997–2006									
Cementless modular #1	1,632	774	99 (98–99)	60	97 (96–98)	0	–	0.69 (0.24–1.99)	0.5
Cementless modular #2	534	334	99 (98–100)	19	–	0	–	0.68 (0.21–2.23)	0.5
Cemented THR	176	93	98 (96–100)	3	–	0	–	1.0	–
Total	3,668								

A–J See Table 3.

### Survival of stem groups, aseptic loosening

**Figure 3.**
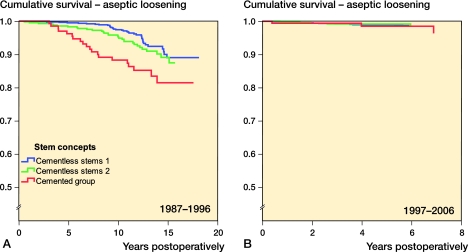
Cox-adjusted survival curves of 3,668 stems in patients aged less than 55 years with stem group as the strata factor. The endpoint was defined as stem revision due to aseptic loosening. Adjustment was made for age and sex. A. THRs performed 1987–1996. Cementless stem group 1 had a significantly better overall survival than the reference group of cemented stems. B. THRs performed 1997–2006. The differences in survival rates between the stem groups were not statistically significant.

In the Cox regression analysis, cementless stem group 1 operated 1987–1996 had a lower risk of revision than the cemented stem group operated during the same time period (RR = 0.4; CI: 0.23–0.68) (p = 0.001) (Table 5 and Figure 2A). The revision risk of cementless stem group 2 operated 1987–1996 was lower than that of the cemented stems operated during the same time period (RR = 0.6; CI 0.32–1.00) (p = 0.05) (Table 5 and Figure 3).

**Table 5.** Survival of stem groups. The endpoint was defined as revision due to aseptic loosening of the stem. 5-, 10-, and 15-year survival rates were obtained from the Kaplan-Meier analysis

A	B	C	D	E	F	G	H	I	J
1987–1996									
Cementless stem #1	767	717	100 (99–100)	586	97 (96–99)	106	89 (85–93)	0.39 (0.23–0.68)	0.001
Cementless stem #2	419	396	98 (97–100)	321	95 (93–97)	76	89 (85–93)	0.57 (0.32–1.00)	0.05
Cemented stem	140	128	96 (93–100)	105	88 (83–100)	33	81 (73–89)	1.0	–
1997–2006									
Cementless stem #1	1,632	773	99 (98–100)	60	98 (98–99)	0	–	0.63 (0.18–2.16)	0.5
Cementless stem #2	534	334	99 (98–100)	19	–	0	–	0.49 (0.12–2.05)	0.3
Cemented stem	176	93	99 (96–100)	3	–	0	–	1.0	–
Total	3,668								

A Stem group cementless stem # 1: a cementless, straight proximally porous-coated stem. cementless stem # 2: a cementless, anatomic proximally porous- and/or hydroxyapatite-coated stem. B–H and J See Table 3. I Risk ratio of revision (95% CI) from the Cox regression analysis (other stem groups were compared with the cemented stems; adjustment made for age and sex).

### Survival of cup groups, aseptic loosening

**Figure 4.**
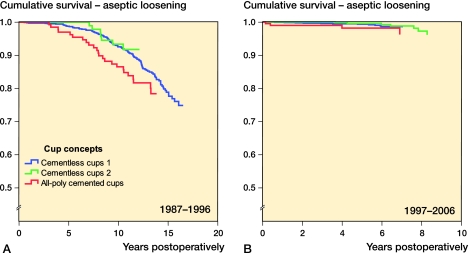
Cox-adjusted survival curves of 3,668 cups in patients aged less than 55 years with cup group as the strata factor. The end point was defined as cup revision due to aseptic loosening. Adjustment was made for age and sex. A. THRs performed 1987–1996. The differences in survival rates between the cup groups were not statistically significant. B. THRs performed 1997–2006. The differences in survival rates between the cup groups were not statistically significant.

In the Cox regression analysis, the differences in revision rates between groups were not statistically significant (Table 6 and Figure 4). The 10-year survival of cementless group 1 operated 1997–2006 was better than that of the same group operated 1987–1996 (Table 6).

**Table 6.** Survival of cup groups. The endpoint was defined as revision due to aseptic loosening of the cup. 5-, 10-, and 15-year survival rates were obtained from the Kaplan-Meier analysis

A	B	C	D	E	F	G	H	I	J
1987–1996									
Cementless cup #1	1,075	1,010	99 (98–99)	831	92 (91–94)	183	79 (75–82)	0.79 (0.52–1.22)	0.3
Cementless cup #2	111	105	100 (100–100)	82	94 (88–99)	2	–	0.69 (0.32–1.50)	0.4
Cemented cup	140	127	97 (94–100)	105	86 (80–93)	33	76 (68–85)	1.0	–
1997–2006									
Cementless cup #1	1,512	723	99 (99–100)	58	98 (97–99)	0	–	0.40 (0.13–1.25)	0.1
Cementless cup #2	654	385	100 (99–100)	20	96 (92–99)	0	–	0.45 (0.13–1.54)	0.2
Cemented cup	176	93	98 (96–100)	3	–	0	–	1.0	–
Total	3,668								

A Cup group cementless cup # 1: a cementless, press-fit porous-coated cup. cementless cup # 2: a cementless hydroxyapatite-coated cup. B–H and J See Table 3. I Risk ratio of revision (95% CI) from the Cox regression analysis (other cup groups were compared with the all-polyethylene cemented cups; adjustment made for age and sex)

## Discussion

We found that the survival of cementless THRs was no different from that of cemented THRs in patients under the age of 55 when revision for any reason served as the endpoint. However, excessive wear of the polyethylene liner resulted in numerous revisions of modular cementless cups. After 10 years of follow-up, the revision risk in both cementless groups was higher than that in the cemented reference group. The data from our previous study included years 1980–2001 whereas the current study included years 1987–2006. The overall results in the 2 studies were similar.

This registery-based study has certain limitations. Before 1994, 10% of total hip replacements were missing from the Finnish Arthroplasty Register (Puolakka et al. 2001). These total hip replacements that were lost to follow-up could have been failures and may have caused bias in our study. It is also possible that few centers performed the majority of the replacements, and certain complications could have occurred more often at certain centers. The number of total hip replacements performed for patients under the less than age of 55 years of age with osteoarthritis is considerably lower than the number of replacements performed for elderly patients (Mäkelä et al. 2008). However, the number of hospitals performing cementless replacements for young patients in the current study was high. The total number of cemented implants was low. The data ofn cement brands used have been included in the Finnish Register since 1996. We excluded cement brands with known poor results, such as Boneloc and CMW, from the study to eliminate the effect of cement on the survival of cemented implants. This further reduced the number of cemented THRs. The cement brands were used in 1987–1995 are not known.

A limitation of most register-based studies is that only a revision operation is considered as a definition of failure. There might be patients with polyethylene failure and osteolysis who are not even aware of the problem. In this respect, however, younger patients are probably better off than elderly patients, who might be too ill to undergo revision surgery or who simply prefer not to do so. It is more common to perform revision surgery when needed for younger patients with a long lifetime expectation than for elderly patients. In this respect, a revision operation may be considered as a reasonable definition of failure in a register-based study for patients under the age of 55 years.

Porous-coated cementless cups implanted during the period 1997–2006 had better 10-year survival regarding aseptic loosening than those implanted during 1987–1996. However, 10-year survival for any reason of the later cohort of cementless group 1 was no better than that of the earlier cohort of the same group. This finding is worrisome, because it seems that although cementless cups heal well with bony ingrowth, modern liner options have not provided anything new compared to the older ones regarding the problem of wear and osteolysis. However, the number of implants at risk in 10 years in the later cohort was small compared to the earlier one.

The main concern of patients with long life expectancy and of patients who are younger and more active is the longevity of their total hip arthroplasty. Previous studies have found that the risk of revision is indeed higher in younger patients than in older ones (Herberts and Malchau 2000, Furnes et al. 2001). A good 10-year survival rate of ≥ 90% (NICE) has been recorded for some cementless THAs within patients underbelow 55 years of age, although many of these reports have been from highly specialized clinics and refer to only one brand of implant (Kim et al. 2002, 2003, McAuley et al. 2004, Pieringer et al. 2006, Delaunay et al. 2008, Reigstad et al. 2008, Garcia-Rey et al. 2009, Anseth et al. 2010). Excellent survival results have also been reported using cemented implants for young, high-demand patients with higher physical demands (SHAR 2007, Lewthwaite et al. 2008).

In the present study, survival of cementless stems with aseptic loosening as the end point was superior to that of cemented stems in patients under the age of 55 years. However, the large number of wear-related revisions emphasizes the need for more wear-resistant articulations for cementless cups. For a single patient, each reoperation—including exchange of liner—is a major incident. Therefore, revisions for all reasons should be emphasized in survival analyses. Although there have been reports of the complications associated with ceramic-on-ceramic (Yoon et al. 1998, Park et al. 2006), metal-on-metal (Korovessis et al. 2006, Grübl et al. 2007) and highly cross-linked polyethylene (Bradford et al. 2004) bearings, these alternative bearings have demonstratedshown at least early clinical success with minimal evidence of osteolysis and wear (Callaghan et al. 2008). However, longer follow-up of cementless replacements with alternative bearings is needed.

In conclusion, the survival of cementless THRs was no different from that of cemented THRs when revision for any reason served as the endpoint. However, excessive wear of the polyethylene liner resulted in numerous revisions of modular cementless cups. The population-based outcomes of total hip arthroplasty appeared to be relatively unsatisfactory for younger patients in Finland.

## Appendix

**Table:** Appendix. Implant brands and bone cements used

A	B	C	D	E	F	G	H
Biomet Bimetric	ABG I	Biomet PFU	ABG I	ABG I	Lubinus IP	Lubinus Std	Palacos C Genta
Synergy	ABG II	Biomet Vision	ABG II	ABG II	Lubinus SP I	Müller Std	Simplex Antib
Profile porous	Anatomic Mesh	Harris-Galante II	PCA Pegged	Omnifit Trident	Lubinus SP II	Exeter All-poly	Simplex P
Bicontact	PCA Std	Biomet 38	PCA Vitalock		Exeter Universal	Exeter Contemporary	Palacos R+G
Biomet Integral Lat	PCA E-eries	Biomet M2A	PCA cluster		Müller Monolog	Charnley LPW	Simplex
Biomet Head-Neck	PCA Meridian	Biomet Recap	Omnifit Trident		Charnley	Brunswik	Simplex/Palacos
Proxiloc		Biomet Mallory			Brunswik	Lubinus Eccentric	Palacos
		Trilogy			Spectron	Reflection All-poly	Palacos R Genta
		Biomex					Simplex
		Pinnacle					Tobramy
		Profile Duraloc					
		Bicontact					
		BHR					
		Hedrocel					
		ASR					

A Cementless stems #1 B Cementless stems #2 C Cementless cups #1 D Cementless cups #2 E HA-coated cups F Cemented stems G Cemented cups H Bone cements
